# The HIF/PHF8/AR axis promotes prostate cancer progression

**DOI:** 10.1038/oncsis.2016.74

**Published:** 2016-12-19

**Authors:** D Tong, Q Liu, G Liu, W Yuan, L Wang, Y Guo, W Lan, D Zhang, S Dong, Y Wang, H Xiao, J Mu, C Mao, J Wong, J Jiang

**Affiliations:** 1 Department of Urology, Institute of Surgery Research, Daping Hospital, Third Military Medical University, Chongqing, PR China; 2 Department of Ultrasound, Southwest Hospital, Third Military Medical University, Chongqing, PR China; 3 Department of Bio-Medical Sciences, Philadelphia College of Osteopathic Medicine, Philadelphia, PA, USA; 4 Sbarro Institute for Cancer Research, Department of Biology, College of Science and Technology, Temple University, Philadelphia, PA, USA; 5 Department of Pathology, Institute of Surgery Research, Daping Hospital, Third Military Medical University, Chongqing, PR China; 6 Shanghai Key Laboratory of Regulatory Biology, Institute of Biomedical Sciences and School of Life Sciences, East China Normal University, Shanghai, PR China

## Abstract

Recent studies provide strong evidence that the androgen receptor (AR) signaling pathway remains active in castration-resistant prostate cancer (CRPC). However, the underlying mechanisms are not well understood. In this study, we demonstrate that plant homeo domain finger protein 8 (PHF8 )interacts with and functions as an essential histone demethylase activity-dependent AR coactivator. Furthermore, we demonstrate that the expression of PHF8 is induced by hypoxia in various prostate cancer cell lines. Knockdown of either hypoxia-inducible factor HIF2α or HIF1α almost completely abolished hypoxia-induced PHF8 expression. Importantly, we observed that PHF8 is highly expressed in clinical androgen deprived prostate cancer samples and expression of PHF8 correlates with increased levels of HIF1α and HIF2α. Moreover, elevated PHF8 is associated with higher grade prostate cancers and unfavorable outcomes. Our findings support a working model in which hypoxia in castrated prostate cancer activates HIF transcription factors which then induces PHF8 expression. The elevated PHF8 in turn promotes the AR signaling pathway and prostate cancer progression. Therefore, the HIF/PHF8/AR axis could serve as a potential biomarker for CRPC and is also a promising therapeutic target in combating CRPC.

## Introduction

Prostate cancer is the most frequently diagnosed cancer and the second leading cause of cancer-related death in men in the United States.^1^ Androgen-deprivation therapy remains the mainstream treatment for both locally advanced and metastatic prostate cancers. Unfortunately, although the majority of patients are initially responsive to androgen-deprivation therapy, most tumors eventually progress from hormone-dependent prostate cancer to castration-resistant prostate cancer (CRPC).^2^ Importantly, recent studies indicate that the androgen receptor (AR) still has a pivotal role even in CRPC.^3^ Multiple mechanisms have been proposed to explain the role of AR in androgen-deprivation conditions, including enhanced local synthesis of androgens, increased levels of AR due to upregulated transcription and/or translation, AR mutations and alterations in regulatory factors such as coactivators and corepressors.^4^ Understanding the functions of the AR signaling pathway in CRPC has led to the development of next-generation AR antagonists for CRPC therapy.^5^ Despite these advancements, CRPC is still the major cause of prostate cancer-related death in men.

It has been reported that the degree of tumor hypoxia positively correlates with prostate cancer progression and poor clinical outcomes.^6^ Previous studies have also shown increased hypoxia-inducible factor 1α (HIF1α) gene expression in prostate cancer tissues.^7^ Furthermore, hypoxia has been shown to increase AR transcriptional activity in prostate cancer cells.^8, 9^ These observations could partially explain why inhibiting HIF1α attenuates AR signaling pathways and represses tumor progression in CRPC.^10^ Castration induced local prostate hypoxia was initially observed in animal models^11^ and recent studies have provided evidence that both chemical and surgical castration treatments for patients with hormone-dependent prostate cancer are also associated with local hypoxia and subsequent activation of the HIF pathway.^11^ Thus, it is of critical significance to elucidate the underlying mechanisms by which castration-induced hypoxia promotes AR activation and the development of CRPC.

Plant homeo domain finger protein 8 (PHF8), also known as Jumonji domain-containing histone demethylase, is a member of the histone demethylase family. Numerous studies collectively show that PHF8 is capable of demethylating mono- and di-methylated histone H3 lysine 9 (H3K9me1/2), di-methylated histone H3 lysine 27 (H3K 27me2), mono-methylated histone H4 lysine 20 (H4K20me1) and possibly di-methylated histone H3 lysine 36 (H3K36me2).^12, 13, 14, 15, 16^ Consistent with its histone demethylase activity, PHF8 has been demonstrated to promote transcriptional activation of various Pol II-transcribed genes and ribosomal DNA transcription by RNA polymerase I.^17, 18^ Consistent with the finding that PHF8 mutations are loosely linked with X-linked mental retardation, PHF8 was also shown to function as a coactivator for retinoic acid receptor and has a role in neural differentiation.^19^ Furthermore, PHF8 was observed to be highly expressed in cancers, including non-small cell lung cancer, esophageal squamous cell carcinoma, acute promyelocytic leukemia, cervical cancer and prostate cancer.^15, 20, 21, 22, 23, 24, 25^ A recent study reported that PHF8 promotes prostate cancer cell growth by activating miR-125b.^26^ However, the underlying mechanism for enhanced PHF8 expression in prostate cancer is unknown. Furthermore, the functional relationship between PHF8 and the AR signaling pathway and prostate cancer progression following castration treatment remain poorly understood.

In this study, we demonstrate that PHF8 interacts with and functions as a coactivator for the AR. Furthermore, we demonstrate that the expression of PHF8 is induced by hypoxia in prostate cancer cell lines and this induction requires HIF1α and HIF2α. Finally, we provide evidence that the levels of PHF8 in prostate cancer clinical samples correlate with increased Gleason grade, poor prognosis and lower overall survival of prostate cancer patients. We propose that a novel regulatory axis, HIFs/PHF8/AR, exists in prostate cancer and targeting this axis could be a potential therapeutic strategy in combating castration-induced CRPC.

## Results

### PHF8 interacts with and transactivates the AR in a demethylase activity-dependent manner

**Figure 1:**
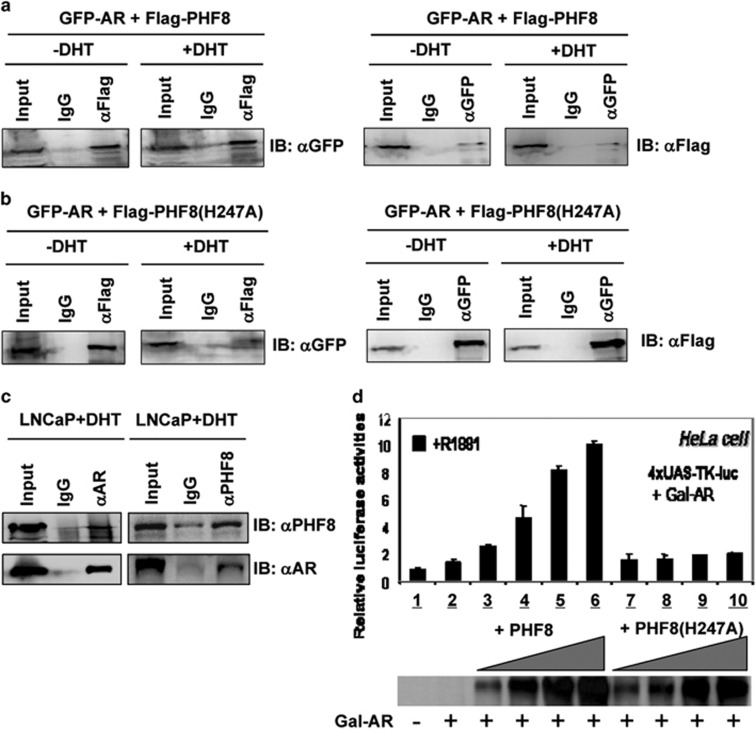
PHF8 interacts with the AR and functions as an AR coactivator. (**a**) PHF8 interacts with AR independent of DHT. HEK293T cells were transiently transfected with plasmids expressing Flag-tagged PHF8 and GFP-tagged AR. Cells were treated with or without 10 nM DHT for 48 h and the cell lysates were used for immunoprecipitation with antibodies against either Flag or GFP. Western blotting was conducted with antibodies as indicated. (**b**) PHF8 interacts with AR independent of its demethylase activity. HEK293T cells were transiently transfected with plasmids expressing Flag-tagged PHF8 (H247A) mutant and GFP-tagged AR. The interaction assay by co-immunoprecipitation was performed as described above. (**c**) In LNCaP cells endogenous PHF8 interacts with AR. LNCaP cells were treated with 10 nM DHT for 48 h and the cell lysates were used for immunoprecipitation with antibodies against either PHF8 or AR. Western blotting was conducted with AR or PHF8 antibody as indicated. (**d**) PHF8 enhances AR transcriptional activation in a demethylase activity-dependent manner. HeLa cells were co-transfected with the 4 × UAS-TK-luc reporter, the plasmids expressing the fusion protein Gal-AR, and increased amount of either the wild-type PHF8 or mutant PHF8 (H247A) as indicated. Cells were cultured with or without 10 nM R1881 overnight. The relative luciferase activities in the cell lysates were normalized to renilla luciferase activity. The relative luciferase activities are presented as mean±s.e.m. of three independent transfections and the levels of PHF8 were estimated by western blot as depicted in the bottom panel.

In previous studies, PHF8 has been shown to interact with and enhance transcriptional activation of the retinoic acid receptor.^22, 27^ Given the reported increased PHF8 expression in prostate cancer clinical samples,^24^ we tested if PHF8 also interacts with the AR. We co-expressed a green fluorescent protein (GFP)-tagged AR and Flag-tagged PHF8 in 293FT cells and treated the transfected cells with or without the AR agonist dihydrotestosterone (DHT) for 24 h. Co-immunoprecipitation was then conducted with antibodies against Flag or GFP. As shown in Figure 1a left panel, GFP-AR was detected when Flag-PHF8 was immunoprecipitated with anti-Flag antibody, but not the control Immunoglobulin G (IgG), and this co-immunoprecipitation of GFP-AR with Flag-PHF8 is independent of DHT treatment. Similarly, Flag-PHF8 was detected when GFP-AR was immunoprecipitated with anti-GFP antibody in a DHT-independent manner (Figure 1a, right panel). To test if demethylase activity is required for interaction with the AR, we carried out co-immunoprecipitation experiments with the demethylase-deficient mutant PHF8 (H247A). As shown in Figure 1b, the PHF8 (H247A) mutant interacted with AR, indicating that the demethylase activity of PHF8 is unnecessary for PHF8-AR interaction.

To test if endogenous PHF8 and AR interact with each other, we prepared whole-cell extracts from the AR positive prostate cancer cell line, LNCaP. We detected endogenous PHF8 when AR was immunoprecipitated with anti-AR antibody in DHT-treated LNCaP cells (Figure 1c). In addition, endogenous AR was detected when PHF8 was immunoprecipitated with an anti-PHF8 antibody in DHT-treated LNCaP cells, further supporting the interaction between endogenous PHF8 and AR in LNCaP cells.

Having demonstrated that PHF8 interacts with AR, we next examined if PHF8 regulates AR transcriptional activity. Toward this end, a luciferase reporter assay was employed. We transfected a 4xUAS-TK-luc reporter, a plasmid expressing a fusion protein of Gal4 DNA-binding domain and AR (Gal-AR), and various concentrations (as indicated) of plasmids encoding the wild-type or H247A mutant PHF8 into 293FT cells (Figure 1d). The cells were treated with the synthetic AR agonist R1881 for 24 h before the cells were harvested for luciferase activity assay. As shown in Figure 1d, co-expression of PHF8 enhanced AR transcriptional activation in a dose-dependent manner. This coactivator activity is dependent on its demethylase activity as it was not observed in the PHF8 (H247A) mutant (Figure 1d). This difference in coactivator function between the wild-type and mutant PHF8 was not due to difference in protein expression as revealed by western blot analysis (Figure 1d, lower panel). Thus, the reporter assay provides evidence that PHF8 functions as an AR coactivator and does so requiring its demethylase activity.

### PHF8 is recruited to and required for optimal transcriptional activation of AR target genes

**Figure 2:**
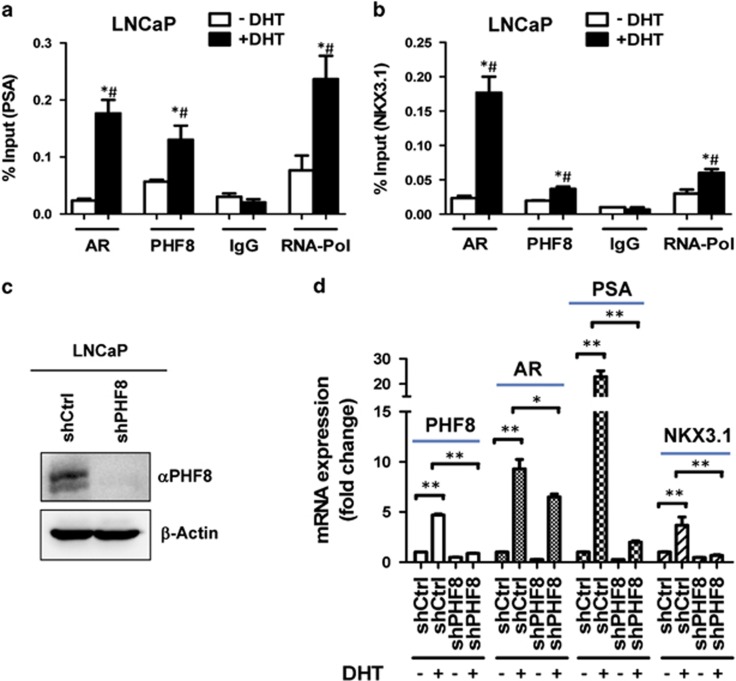
PHF8 is recruited to AR target gene promoters and is essential for optimal hormone-dependent transcriptional activation of AR target genes. (**a**, **b**) PHF8 is recruited to the AR target genes PSA (**a**) and NKX3.1 (**b**) upon DHT treatment in LNCaP cells. LNCaP cells were cultured in absence or presence of DHT for 48 h. Whole-cell lysates were subjected to ChIP assays with nonspecific IgG or antibodies against AR, PHF8 or RNA polymerase II (RNA-Pol). Purified DNA was used for qPCR with primers targeting the promoter regions of the PSA (Left Panel) and NKX3.1 (Right Panel). The mean values±s.e.m. were shown. Student's *t-*tests were performed to compare the assays with specific antibodies against AR, PHF8 and RNA-Pol, or nonspecific IgG. *P<0.05, compared with DHT-negative group; and ^#^*P*<0.05, compared with IgG group. (**c**) Western blot analysis showing knockdown of PHF8 by shRNA against PHF8. LNCaP cells were infected with a lentiviral shPHF8 and western blot analysis was carried out with an anti-PHF8 antibody. (**d**) Knockdown PHF8 severely impaired DHT-induced transcriptional activation of PSA and NKX3.1. The control shRNA and shPHF8 infected LNCaP cells were treated with 10 nM DHT for 24 h and total RNAs were prepared and subjected to quantitative RT–PCR analysis. The results are presented as the mean±s.e.m. of three independent experiments.

We next analyzed the role of PHF8 in AR transcriptional activation in LNCaP cells. We first tested if PHF8 were recruited to AR target genes upon DHT-induced AR transcriptional activation. To this end, a chromatin immunoprecipitation (ChIP) assay was performed to examine the association of PHF8 with two well characterized AR target genes, PSA and NKX3.1. As shown in Figure 2a, ChIP assays revealed that, as expected, DHT treatment resulted in significantly increased binding of AR and elevated levels of RNA pol II at the PSA promoter. DHT treatment also resulted in elevated association of PHF8 with the PSA promoter. Similarly, DHT treatment not only increased the binding of AR and recruitment of pol II at the NKX3.1 gene promoter, but also resulted in an increased occupancy of PHF8 at the NKX3.1 promoter (Figure 2b). Thus, consistent with a protein–protein interaction with AR, our ChIP analysis demonstrated that PHF8 is recruited to AR target genes upon DHT treatment.

We then examined the role of PHF8 in DHT-induced transcriptional activation of AR target genes. We first established conditions to knockdown PHF8 in LNCaP cells using short hairpin RNA (shRNA) against PHF8 (Figure 2c). Quantitative reverse transcriptase (RT)–PCR analysis revealed an ~60% downregulation of the PHF8 mRNA (Supplementary Figure 1). We then tested the effect of PHF8 knockdown on DHT-induced transcriptional activation of PSA and NKX3.1 in LNCaP cells by quantitative RT–PCR. As shown in Figure 2d, knockdown of PHF8 significantly impaired DHT-induced transcriptional activation of PSA and NKX3.1 genes. Knockdown of PHF8 also reduced the DHT-induced transcriptional activation of AR, although to a lesser extent in comparison to PSA and NKX3.1. Interestingly, we found that the expression of PHF8 was also induced by DHT treatment and this induction could be abolished by PHF8 knockdown. Similarly, we observed that PHF8 mRNA was also induced by DHT treatment in VCaP prostate cancer cell line. Furthermore, we found that knockdown of PHF8 in VCaP cells also impaired DHT-induced expression of AR target genes PSA and NKX3.1 (Supplementary Figure 2). We also noticed that knockdown of PHF8 only had a marginal effect on AR levels, possibly due to a very strong expression of AR in VCaP cells. Together, these data indicate that PHF8 is not only recruited to but is also required for optimal transcriptional activation of AR target genes. Based on the observed protein–protein interaction between PHF8 and AR, we suggest that PHF8 recruitment to AR target genes is likely due to its interaction with AR.

### Hypoxia induces PHF8 expression in prostate cancer cells

**Figure 3:**
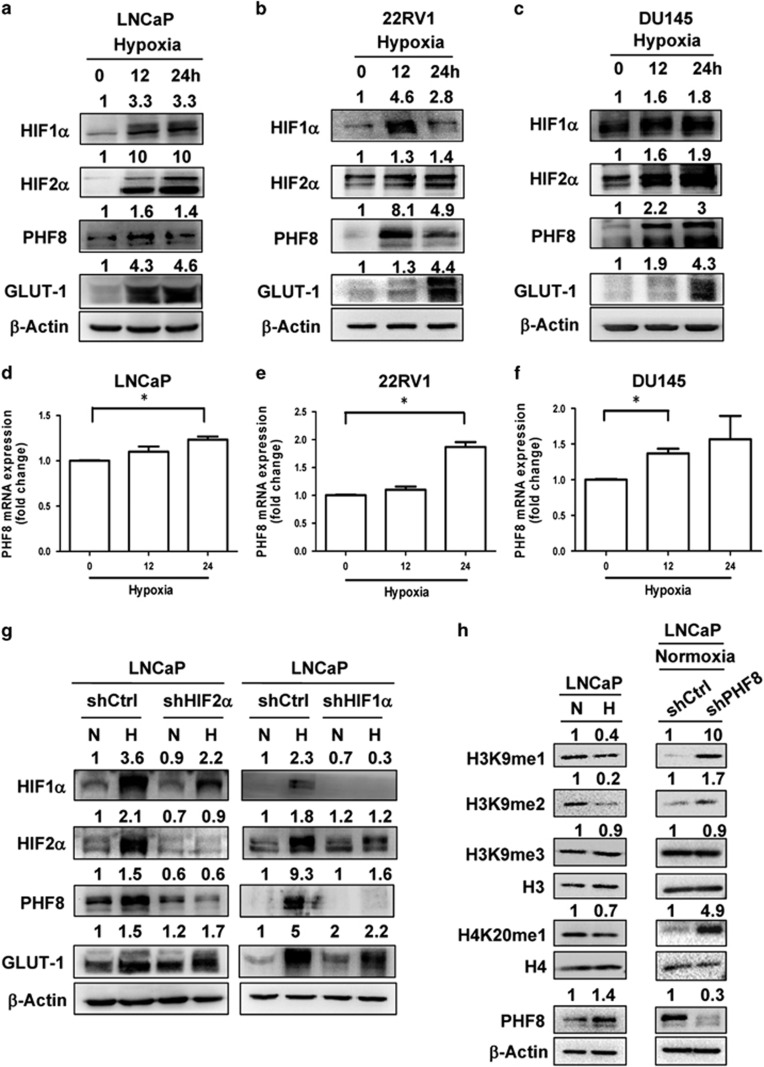
PHF8 is upregulated by HIFs during hypoxia and regulates histone methylation in response to hypoxia. (**a**–**c**) LNCaP (**a**), 22RV1 (**b**) and DU145 (**c**) cells were cultured first at normoxic conditions and then shifted to hypoxic conditions for 0, 12, 24 h. Cells were collected and whole-cell lysates were prepared. Proteins were separated on 8% SDS–polyacrylamide gel electrophoresis and western blotting was performed with antibodies against HIF1α, HIF2α, PHF8, GLUT-1 and β-actin as indicated. (**d**–**f**) LNCaP (**d**), 22RV1 (**e**) and DU145 (**f**) cells were cultured first at normoxic conditions and then shifted to hypoxic conditions for 0, 12, 24 h. The total RNAs were then prepared and quantitative RT–PCR for PHF8 was performed. The results are presented as the mean±s.e.m. of three independent experiments. (**g**) Knockdown HIF2α or HIF1α abrogated hypoxia-induced upregulation of PHF8 proteins. LNCaP cells stably infected with control shRNA (shCtrl) or shHIF2α (left panel) /shHIF1α (right panel) were cultured under normoxic (N) or hypoxic (H) conditions for 12 h and then collected for western blot analysis using antibodies as indicated. (**h**) Hypoxia-induced PHF8 expression correlates with reduced levels of H3K9me1/2 and H4K20me1. On the left panel, LNCaP cells were subjected to normoxic and hypoxic conditions for 12 h. Whole-cell lysates were prepared and proteins were separated on SDS–polyacrylamide gel electrophoresis. Then western blots were conducted with antibodies against H3K9me1, H3K9me2, H3K9me3, H4K20me1, H3, H4, PHF8 and β-actin as indicated. On the right panel, LNCaP cells were stably infected with control shRNA or shPHF8 and the histone methylation status under normoxic conditions were analyzed by western blot. Note that PHF8 knockdown resulted in increased levels of H3K9me1/2 and H4K20me1 but not H3K9me3, indicating that PHF8 controls global levels of H3K9me1/2 and H4K20me1 in LNCaP cells. The quantitative analysis of western blot results was based on Image J software version 1.44p (NIH, USA).

Recent studies have provided evidence for hypoxia in prostate cancer tissues, especially upon castration.^7, 28, 29^ Since increased PHF8 expression has been reported to occur broadly in prostate cancer, we hypothesized that increased PHF8 expression in prostate cancer could be at least partly induced by hypoxia. To test this hypothesis, we cultured three prostate cancer cell lines, LNCaP, 22RV1 and DU145 under hypoxic conditions for 0, 12 and 24 h and then examined the levels of PHF8 protein by western blot analysis and mRNA by RT–PCR. As shown in Figures 3a–c, we found that hypoxia resulted in elevated levels of PHF8 protein in all three types of cells. We also observed that HIF1α, HIF2α and glucose transporter 1 (GLUT-1), a well-established HIF1α target gene,^30^ were induced by hypoxia, thus verifying our hypoxic conditions. RT–PCR analysis demonstrated that hypoxia treatment elevated the levels of PHF8 mRNA (Figures 3d–f). These results demonstrate that PHF8 mRNA can be induced by hypoxia. However, since the increase in PHF8 mRNA did not always match well with the increase in PHF8 protein expression, it is possible that hypoxia may also regulate PHF8 protein stability or translation.

To examine if hypoxia-induced PHF8 expression is dependent on HIF family transcription factors, we first knocked down HIF1α or HIF2α in LNCaP cells with their specific shRNAs and then subjected the cells to normoxic or hypoxic conditions for 12 h. Subsequent western blot analysis revealed that knockdown of either HIF1α or HIF2α impaired hypoxia-induced PHF8 expression (Figure 3g). We, therefore, conclude that hypoxia-induced PHF8 expression is dependent on expression of either HIF1α or HIF2α.

### Hypoxia-induced PHF8 expression alters global histone methylation

Having established that hypoxia induced PHF8 expression in prostate cancer cells, we next determined if PHF8 regulates histone methylation in prostate cancer cells. To this end, we first compared the global levels of H3K9me1, H3K9me2 and H4K20me1 which are known substrates for PHF8, by western blot analysis. As shown in Figure 3h left panel, hypoxia induced PHF8 expression and resulted in a concomitant global reduction of H3K9me1, H3K9me2 and H4K20me1. Hypoxia had no effect on the level of H3K9me3, which is not a substrate for PHF8, suggesting that the observed changes in H3K9me1, H3K9me2 and H4K20me1 are likely due to increased PHF8 expression. Since hypoxia has also been shown to induce the expression of other histone demethylases, including JMJD1A JMJD2B JMJD2C,^31, 32^ we also analyzed the effect of PHF8 knockdown on global histone methylation in LNCaP (Figure 3h, right panel) and PC-3 cells (Supplementary Figure 3) under normoxic conditions. As shown in Figure 3h right panel, knockdown of PHF8 resulted in elevated levels of H3K9me1/2 and H4K20me1 but did not increase H3K9me3. These results support an important role for PHF8 in control of global histone methylation in LNCaP and PC-3 cells.

*In silico* analysis found four potential HIF-binding sites with core sequences of GCGTG or ACGTG (Supplementary Figure 4A) within the region of −633 to −168 bp of the human PHF8 promoter. To investigate if HIF transcription factors can directly regulate PHF8 expression through these potential HIF-binding sites, we constructed luciferase reporters with or without these sequences (Supplementary Figure 4B). Subsequent luciferase reporter assays in 293FT cells showed that both HIF1α and HIF2α strongly induced the transcription of the luciferase reporter with the −1281 to +1 region of PHF8 promoter but not the luciferase reporter with −33 to +1 region of the PHF8 promoter (Supplementary Figure 4C–D). The reporter assay thus supports the idea that HIF transcription factors could bind to PHF8 proximal promoter and activate PHF8 transcription under hypoxic conditions.

### PHF8 is functional under hypoxic conditions

**Figure 4:**
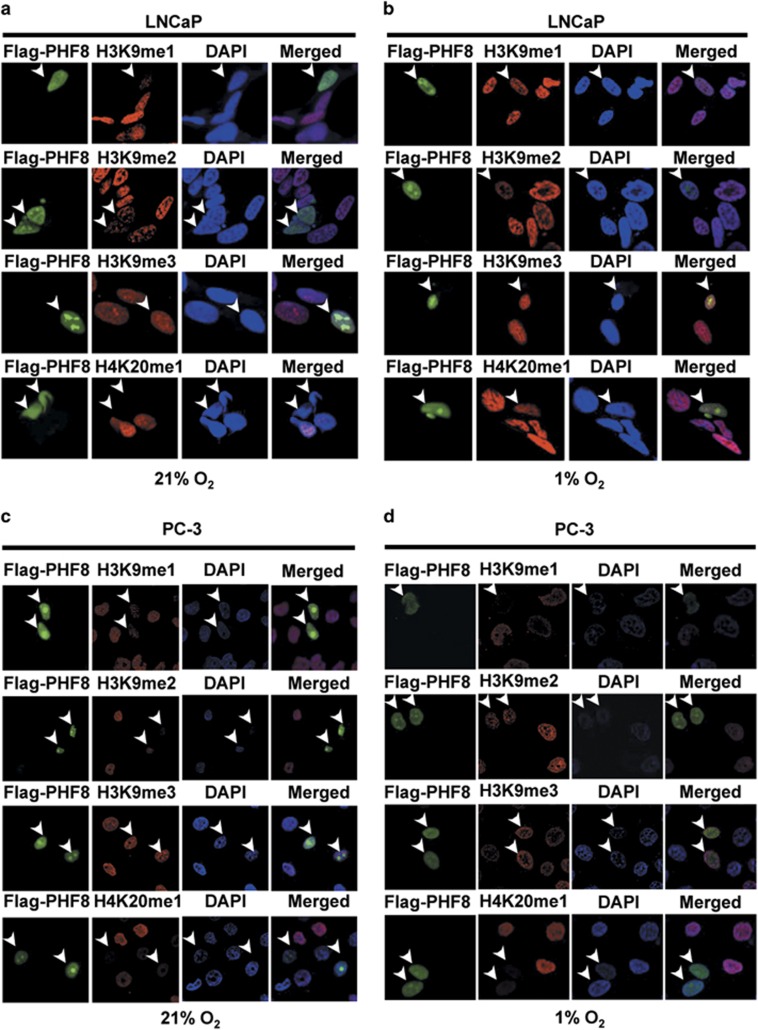
PHF8 is functional under both normoxic and hypoxic conditions. Flag-PHF8 was transfected into LNCaP (**a**, **b**) and PC-3 (**c**, **d**) cells and 48 h after transfection the cells were cultured in normoxic (**a**, **c**) or hypoxic (**b**, **d**) conditions for additional 12 h. The cells were then subjected to double immunofluorescent staining analysis for Flag-PHF8 and a methylated histone as indicated.

As a member of the JmjC-containing demethylase family, PHF8-catalyzed histone demethylation depends on the availability of O_2_.^33^ Previous studies showed that the effect of oxygen concentrations on the hydroxylation-dependent demethylation activity is enzyme specific.^9, 32^ For example, JMJD2A and JARID1A are inhibited by severe hypoxia, but JMJD1A was not. Since demethylases have different sensitivities to the levels of O_2_,^9^ we were interested in understanding whether PHF8 is functional under severe hypoxic conditions. Toward this end, we transiently transfected LNCaP (Figures 4a and b) and PC-3 (Figures 4c and d) cells with plasmids coding for PHF8 and determined the ability of PHF8 overexpression to demethylate histones under both normoxic (21% O_2_) and hypoxic (1% O_2_) conditions by immunofluorescent staining. The results showed that PHF8 overexpression in two cell lines (indicated by arrows) correlated with reduced levels of H3K9me1, H3K9me2 and H4K20me1, but not H3K9me3 under both conditions (Figures 4a–d). These results demonstrated that as a histone demethylase PHF8 is functional under both normoxic and hypoxic conditions.

### Elevated levels of PHF8 in prostate cancer correlate with hypoxia

**Figure 5:**
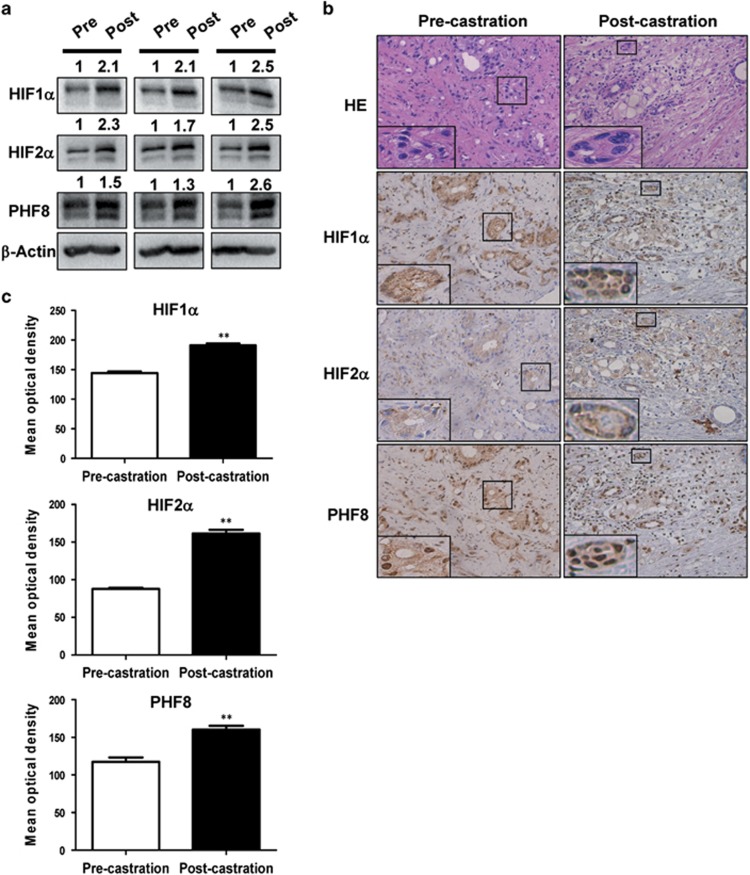
Castration-induced hypoxia upregulates HIFs and PHF8 in prostate cancer tissues (**a**) Western blotting was conducted on samples from pre-and post-castration treatment in the same patients, using antibodies against HIF1α, HIF2α and PHF8 and β-actin. The intensities of the bands were estimated by Image J and normalized by comparing to that of the β-actin. Results from three representative patients were shown. (**b**) The representative results of immunohistochemistry analysis on pre- and post-castration prostate cancer tissues. The pre- and post-castration prostate cancer samples from 14 prostate cancer patients were stained with antibodies against HIF1α, HIF2α and PHF8. Original magnification was × 400. (**c**) The staining intensities were semi-quantified and the averages of the optical densities are shown in the bar graphs. Student's *t*-tests were used to compare differences between two groups, **P<0.01.

Having demonstrated that hypoxia can induce PHF8 overexpression in prostate cancer cell lines,^24^ we hypothesized that hypoxia in prostate cancer could be a mechanism for the increased PHF8 expression observed in clinical prostate cancer samples. Furthermore, since both chemical and surgical castration treatments for prostate cancer patients have been shown to correlate with local hypoxia and subsequent activation of the HIF pathway,^11^ castration is likely to further induce the expression of PHF8. Consistent with previous reports,^34^ qualitative analyses of color Doppler imaging of transrectal ultrasound found that castration treatment reduced blood flow to the prostate cancer tissues, suggesting local ischemia and hypoxic conditions in prostate cancer tissues (Supplementary Figure 5). To substantiate this observation, we compared the levels of PHF8, HIF1α and HIF2α in pre- and post-castration prostate cancer tissues from 14 patients with advanced prostate cancer by western blot analysis. The representative results for three patients are shown in Figure 5a, and the detailed information and the levels of HIF1α, HIF2α and PHF8 in tissue samples pre- and post-castration for all these patients are shown in Supplementary Table 1. The elapsed times between castration and post-castration were about 1–3 months, with an average of 1.5 months. Collectively these results indicate that the levels of PHF8, HIF1α and HIF2α were all elevated in the post-castration cancer tissues (Figure 5a). To further substantiate this observation, we performed immunohistochemistry (IHC) staining on formalin-fixed, paraffin-embedded tissues for 14 pairs of pre- and post-castration prostate cancer samples. The representative results in Figure 5b show that HIF1α, HIF2α and PHF8 were found in both the cytoplasm and nuclei and the levels of these factors are relatively higher in the samples from the post-castration treatment. A Pearson *χ*^2^ test was used to analyze the average levels of HIF1α, HIF2α and PHF8 before and after castration treatment (Supplementary Table 2) and the results in Figure 5c show that castration resulted in elevated levels of these factors. Together, these results suggest that castration treatment can lead to local ischemia/hypoxia in prostate cancer tissues and a concurrent up-regulation of HIF1α and HIF2α as well as PHF8.

### Elevated levels of PHF8 in prostate cancer correlate with higher Gleason grades and poor prognoses

**Figure 6:**
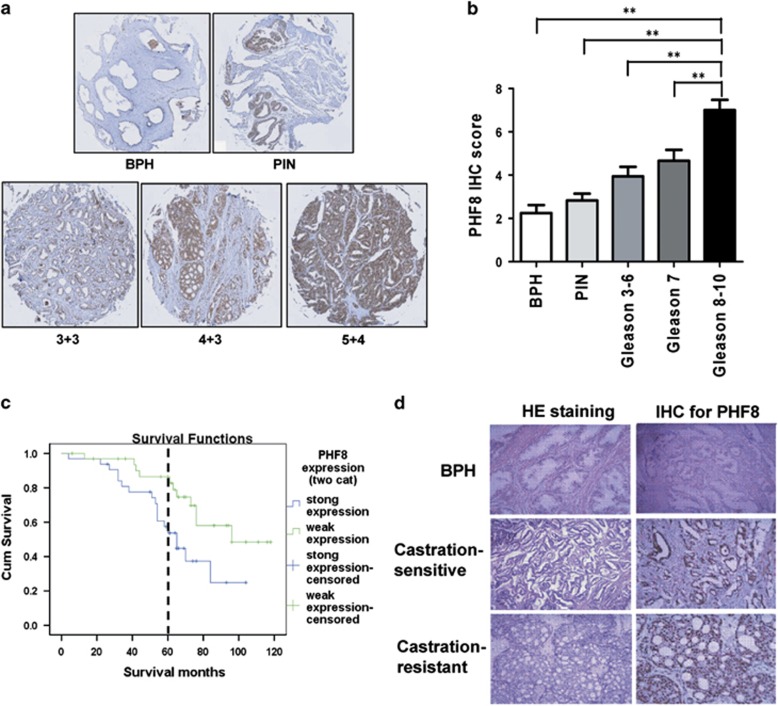
Elevated levels of PHF8 correlate with higher Gleason grades and poor prognosis (**a**) The representative results of immunohistochemistry analysis of PHF8 in benign prostatic hyperplasia (BPH), prostatic intraepithelial neoplasia (PIN) and prostate cancer tissues with different Gleason grades. (**b**) The protein level (mean±s.e.m.) of PHF8 in BPH (*N*=16), PIN (*N*=16) and prostate cancer tissues with Gleason3-6 (*N*=18), Gleason7 (*N*=21) and Gleason8-10 (*N*=26). **P<0.01. (**c**) Five-year (indicated by a dotted vertical line) and overall survival of prostate cancer patients with high- or low-PHF8 scores was shown. Patients with high PHF8 expression (*N*=32; IHC score 6-9) had worse 5-year and overall survival compared with patients with low PHF8 expression (*N*=33; IHC score 1–4) as measured by death or being censored from the study. (**d**) The representative results of HE and IHC staining for PHF8 in human tissues with BPH, castration-sensitive and castration-resistant prostate cancers.

Having established that PHF8 could be induced by hypoxia and function as an AR coactivator, we next wished to examine if PHF8 could be used as a biomarker for prostate cancer. We performed IHC analysis on prostate cancer tissue samples from 97 patients including 16 benign prostatic hyperplasia, 16 prostate intraepithelial neoplasia and 65 prostate cancers that were collected during prostatectomy between 2000 and 2006 at Daping Hospital of Surgery Research (Third Military Medical University, Chongqing, China). The general characteristics of the patients, including age, Gleason scores, PHF8 IHC staining scores and the survival status are presented in Supplementary Table 3. The ages of the patients were between 44 and 74 years (66.4±6.4). The median follow-up time after surgery was about 5 years and the median overall survival of patients was 64 months. Among them, 29 patients died from metastatic prostate cancer. As a first step of a systemic examination of the roles of PHF8 and exploration of the mechanisms in prostate cancer development, we decided to assess the relationship between PHF8 expression and different malignant stages of prostate cancer. Figure 6a shows the representative results of PHF8 staining in benign prostatic hyperplasia, prostate intraepithelial neoplasia and prostate cancer tissues at different malignancy stages (Gleason score 3+3, 4+3 and 5+4). Quantitative analysis of PHF8 expression using Kruskal–Wallis test followed by Dunn's Multiple Comparison Test confirmed that PHF8 is progressively upregulated from benign prostatic hyperplasia to prostate intraepithelial neoplasia to higher Gleason score prostate cancer (Figure 6b). Based on PHF8 immunostaining scores, the 65 prostate cancer patients could be categorized into strong (33 patients) and weak (32 patients) PHF8 groups, respectively. The relationship between the levels of PHF8 and tumor-related variables was analyzed by a Pearson *χ*^2^ test. As shown in Supplementary Table 4, the average ages of patients in two groups were not significantly different according to chi-square statistics (*P*=0.710). However, compared with the weak-PHF8 group, the strong-PHF8 group contained more poorly differentiated patients with Gleason scores 8–10 (*P*<0.001). Moreover, PHF8 displayed more cytoplasmic staining in tumors with lower Gleason grades and tended to exhibit a stronger nuclear staining in tumors with higher Gleason grades (Supplementary Figure 6).

To determine whether the levels of PHF8 in prostate cancer correlate with clinical outcome, we conducted Kaplan–Meier analyses on the follow-up data obtained from these 65 patients. Five-year survival (Figure 6c, indicated by a dotted vertical line, log-rank *P*=0.008) and overall survival (Figure 6c, log-rank *P*=0.016) were significantly lower in patients with strong PHF8 expression (55% and 25%, respectively) than those with weak PHF8 expression (85% and 50%, respectively). Comparing mortality among PHF8-strong patients to that of PHF8-weak patients as the reference, the hazard ratio was 2.5 (95% confidence interval: 1.2–5.4). In addition, the levels of PHF8 in benign prostatic hyperplasia, castration-sensitive and castration-resistant specimens are highly correlated with differentiation grade and the highest level of PHF8 was observed in castration-resistant cancers (Figure 6d). Finally, Pearson *χ*^2^ test also found that the number of patients with higher PHF8 expression is also significantly higher in the castration-resistant group (Supplementary Table 5). These data collectively demonstrated that the levels of PHF8 are highly correlated with malignant stages of prostate cancer and also the 5-year and overall survivals of patients with prostate cancer, suggesting that PHF8 has important roles in prostate cancer progression.

## Discussion

**Figure 7:**
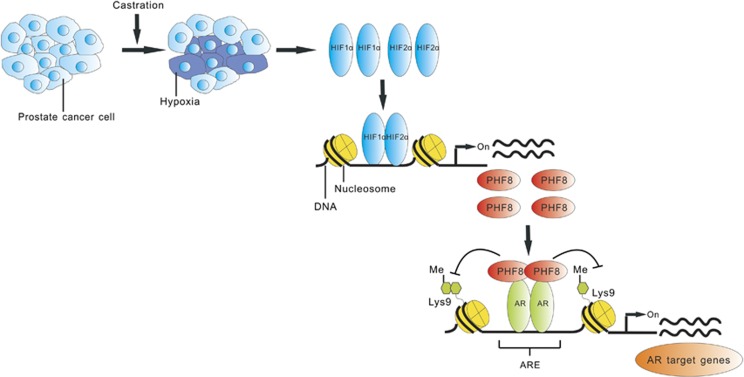
A proposed working model of the HIF/PHF8/AR axis in CRPC progression. Prostate cancer- and castration-induced hypoxia upregulate both HIF1α and HIF2α in prostate cancer cells, which subsequently upregulates PHF8 expression. Under both normoxic and hypoxic conditions, PHF8 interacts with and facilitates AR transcriptional activation. Mechanistically, PHF8 specifically modifies the histone tails through its demethylase activity in the promoter regions of AR target genes and enhances the expression of AR targets. HIFs, PHF8 and AR thus act together to favor the development and/or progression of CRPC.

As a multi-functional histone demethylase, PHF8 has been shown to be overexpressed in various cancers including prostate cancer.^21, 24, 25, 26, 35^ Furthermore, PHF8 has been identified through a systematic screening of epigenetic enzymes as a novel demethylase with an impact on cell proliferation, migration and invasion of prostate cancer cells.^24^ In this study, we provide evidence that PHF8 interacts with and functions as a coactivator for the AR and that PHF8 enhances AR transcriptional activation in a demethylase activity-dependent manner (Figures 1 and 2). Furthermore, we demonstrate that PHF8 expression is induced by hypoxia through HIF transcription factors (Figure 3). Importantly, we provide evidence that the levels of PHF8 in prostate cancer correlates with tumor hypoxia, Gleason grades, poor prognosis and lower overall survival (Figures 5 and 6) and that PHF8 is functional under hypoxic conditions (Figure 4). Altogether, our data suggest the existence of a HIF/PHF8/AR axis that promotes prostate cancer progression and allows us to propose a working model (Figure 7). In this model, we propose that prostate cancer generates a hypoxic microenvironment, which activates HIFs. The activation of HIFs in turn stimulates PHF8 expression. The elevated levels of PHF8 augment AR transcriptional activity and are likely to promote prostate cancer progression at least in part through its ability to promote the AR signaling pathway. However, given its potential role in regulating global histone methylation (Figure 3h), it is also possible that PHF8 could enhance other transcriptional activation processes to promote prostate cancer progression.

The first important finding in our study is that PHF8 functions as an AR coactivator. As a histone demethylase for H3K9me1/2 and H4K20me1, previous studies have linked PHF8 with transcriptional activation for both RNA polymerase I and RNA polymerase II transcribed genes.^17, 18^ The role in transcriptional activation by RNA polymerase II has been linked to its ability to bind H3K4me3 and interact with the C-terminal domain of RNA polymerase II.^18^ In addition, PHF8 has been shown to interact with and act as a coactivator for the retinoic acid receptor.^22, 23^ However, despite of the report that PHF8 is highly expressed in prostate cancer and promotes prostate cancer cell proliferation, migration and invasion,^24^ to our knowledge we are the first to report that PHF8 interacts with AR and functions as an AR coactivator (Figures 1 and 2). Our study thus provides a molecular explanation for the correlation between elevated PHF8 expression and prostate cancer progression.

Another important finding in our study is that hypoxia can induce PHF8 expression through HIF transcription factors (Figures 3 and 5). In this regard, it is noteworthy that we demonstrated that PHF8 is demethylates H3K9me1/2 and H4K20me1 under hypoxic conditions (Figure 4). Thus, different from JMJD2A and JARID1A,^9^ PHF8 functions under both hypoxic and normoxic conditions, suggesting its demethylase activity is less sensitive to the reduced oxygen concentrations in hypoxic prostate cancer. Furthermore, we provide evidence that increased PHF8 expression in prostate cancer clinical samples correlates with the severity of tumor hypoxia (pre- vs post-castration) and elevated levels of HIF1α and HIF2α (Figure 5) and Gleason grades, poor prognosis and lower overall survival (Figure 6). Our results are consistent with the previous studies showing that the extent of tumor hypoxia correlates with prostate cancer progression and poor clinical outcomes^6^ and that both chemical and surgical castration treatments are associated with elevated local hypoxia and activation of the HIF pathway.^11^ We also observe more cytoplasmic staining in tumors with lower Gleason grades and stronger nuclear staining in tumors with higher Gleason grades for PHF8, which is in line with a previous report.^24^ Therefore, our study suggests that the increased PHF8 expression in clinical prostate cancer samples could be at least in part attributed to hypoxia-induced PHF8 transcriptional activation. However, we could not rule out the possibility that hypoxia could also enhance the levels of PHF8 through a post-transcriptional mechanism such as enhanced PHF8 protein stability.

AR amplification and mutation as well as alterations of AR co-activators/co-repressors are believed to be the key drivers of CRPC development.^36^ However, the exact molecular mechanisms for AR activation in CRPC remain ill-defined. In this study, we present evidence that hypoxia induces expression of histone demethylase PHF8. PHF8 interacts with and stimulates AR transcriptional activation through its intrinsic histone demethylase activity. Our study suggests that the HIFs/PHF8/AR axis is likely a driving force for prostate cancer progression and may also be a target for therapy. Given the elevated levels of hypoxia and increased PHF8 expression in CRPCs, targeting PHF8 and/or the HIF1α signaling pathway could be an attractive therapeutic approach in CRPC therapy.

## Materials and Methods

### Antibodies and plasmid constructs

Primary antibodies: Anti-β-Actin (13E5, Cell Signaling Technology, Danvers, MA, USA), Anti-GLUT-1 (ab115730, abcam, Cambridge, MA, USA), Anti-HIF1α (NB100-123, Novus Biologicals, Littleton, CO, USA), Anti-HIF2α(TA301435, Origene, Rockville, MD, USA), Anti-PHF8 (NB100-93314, Novus Biologicals; ab36068, Abcam), Anti-AR(ab133273, Abcam; ab108341, Abcam), Anti-H3 (17168-1-AP, ProteinTech, Rosemont, IL, USA), Anti-H4 (16047-1-AP, ProteinTech), Anti-H3K9me1 (ab8896, Abcam), Anti-H3K9me2 (1349-1, Epitomics, Burlingame, CA, USA), Anti-H3K9me3 (ab8898, Abcam), Anti-H4K20me1 (ab9051, Abcam), Anti-Flag-tag (F1804, Sigma, St Louis, MO, USA), Anti-GFP-tag (66002-1-Ig, ProteinTech), Anti-ER (sc-8002, Santa Cruz Biotechnology, Dallas, TX, USA), and anti-PSA (KG22662-2, KeyGEN BioTECH, Nanjing, PRC). Anti-rabbit secondary antibodies were purchased from Proteintech (SA00001-2) and ComWin Biotech (CW0159, Beijing, PRC). Anti-mouse secondary antibodies were purchased from ComWin Biotech and Proteintech (SA00003-1). Constructs: empty vector, pcDNA3.1-Flag-HIF1α, pcDNA3.1-Flag-HIF2α, pSG5-Flag-PHF8 wild type (WT), pSG5-Flag-PHF8 H247A mutant (MU), pPYCAGIP-GFP-AR, UAS-TK-Luc and Gal-AR were as described.^37, 38, 39, 40, 41^ All plasmid constructs were confirmed by DNA sequencing and by *in vitro* translation and immunoblotting. Cells were transfected using Lipofectamine 2000 (Invitrogen, Grand Island, NY, USA) according to the manufacturer's protocol.

### Patients, tissue samples and IHC

All clinical samples were collected from the Department of Pathology with approval from the Research Ethics Committee of Daping Hospital, Third Military Medical University and written informed consent was obtained from each patient. The sample size was chosen to ensure adequate power based on previous report.^24^ Formalin-fixed, paraffin-embedded tissue specimens were obtained and handled by standard surgical oncology procedure. After deparaffinization and rehydration, the sections were heated in EDTA buffered solution (pH 9.0, ZLI-9069, ZSGB-BIO, China) for 20–40 min in 100 °C boiling water for antigen retrieval. Flanking sections were stained with monoclonal antibodies again HIF1α, HIF2α and PHF8. The secondary antibody conjugated with streptavidin–biotin-horseradish peroxidase complex (Biotinylated Anti-Rabbit IgG (H+L), SP-9001; Biotinylated Anti-Mouse IgG (H+L), SP-9002 (Horseradish Peroxidase Streptavidin, ZSGB-BIO, China). Horseradish peroxidase conjugate used with DAB (3, 3' diaminobenzidine) produced a brown stain (DAB Detection Kit (Streptavidin-biotin, ZLI-9018, ZSGB-BIO, China). Nuclei were lightly counterstained with hematoxylin. All antibodies have been validated for IHC. Negative controls were performed using phosphate-buffered saline without primary antibody. Tissue microarray was scanned by Axio Scan.Z1 (Carl Zeiss Microscopy GmbH system, Germany, Oberkochen, Germany) and analyzed by Zen 2012 (Blue Edition). The HIF1α, HIF2α and PHF8 staining score were assessed by two certified clinical pathologists (Hualiang Xiao and Jianghong Mu) as described previously.^42^ The intensity and extent of scoring of the PHF8 staining were scaled between 0–3. Final scores were computed using a composite of intensity scores multiplied by the extent of staining score. The score of 1~2 was labeled as ‘+', 3~4 as ‘++', and 6~9 as ‘+++'. The score of 1~4 was assessed as weak expression and 6~9 as strong expression. IHC pictures were photographed under light microscope (Olympus, BX53, Tokyo, Japan), and analyzed by Image J software version 1.44p (NIH, USA).

### Survival analysis

Patients were categorized into weak or strong PHF8 groups based on PHF8 levels. Kaplan–Meier survival curves were plotted for survival until death or censored from the study. The 5-year survival and overall survival were shown. *P*-values were calculated by a log-rank test. We used Cox proportional hazard regression to compare mortality of PHF8-strong patients to that of PHF8-weak patients.

### Prostate cancer vascularity by color Doppler imaging of transrectal ultrasound

Transrectal ultrasound examinations were performed using a Sequoia 512 ultrasound scanner (Siemens/Acuson, Mountain View, CA, USA) equipped with a 7-or 10-MHz endorectal probe. The lesions were primarily detected based on gray-scale sonography followed by color Doppler imaging using a low velocity, high-sensitivity color setting for evaluating vascularity signals. A standardized approach was used for each patient with the same color Doppler settings: the velocity scale was set at±0.011 m/s, the wall filter at 1, and the color frequency at 6 MHz. Ultrasound images of prostate cancer tissue were observed and analyzed by professional ultrasound specialist (Yanli Guo) based on blood flow and vascularity.

### Cell culture and treatment

We analyzed five human prostate cancer cell lines, LNCaP, VCaP, 22RV1, DU145 and PC-3 cells and 293FT cells. LNCaP, VCaP, 22RV1 and PC-3 were obtained from the Cell Bank of Shanghai Institutes for Biological Sciences, Chinese Academy of Sciences. All cell lines were authenticated by short tandem repeat (STR) profiling and tested negative for mycoplasma contamination. Cell lines were grown in RPMI medium or high-glucose Dulbecco's Modified Eagle Medium supplemented with 10% heat-inactivated FBS, 10 IU/ml penicillin, and 10 μg/ml streptomycin. Cells were cultured and stored according to the suppliers' instructions. All cells for this study were used within 6 months of resuscitation and cultured at 37 °C in 5% CO_2_ (Thermo, 3131). Hypoxic conditions (1% O_2_, 5% CO_2_ and 94% N_2_) were created in a Forma Series II Water Jacket CO_2_ incubator (model: 3131; Thermo Scientific). For DHT (A8380, Sigma) treatment, cells were preconditioned in hormone-free culture overnight and the final concentration of DHT was 10 nM.

### Generation of HIF1α, HIF2α and PHF8 knockdown cell lines

Control shRNA or shRNAs specific for human HIF1α (SH001530), HIF2α (SH001430) and PHF8 (SH184896) mRNAs were purchased from Yingrun Biotechnology Inc. (Changsha, China) (Supplementary Table 6). LNCaP cells were infected with lentivirus expressing shRNA against human HIF1α, HIF2α and PHF8. Knockdown cell lines were selected with puromycin. Western blot assays were used to detect HIF1α, HIF2α and PHF8 knockdown in LNCaP cells.

### Western blot assays

Protein concentrations were determined by RCDC assay (Bio-Rad, Hercules, California, USA) according to the manufacturer's instructions. Proteins on polyvinylidene difluoride Membranes (PALL, Gelman Laboratory, Ann Arbor, MI, USA) were detected with primary and secondary antibodies as indicated. Detection was performed using ECL western blotting detection reagents (Pierce, Waltham, MA, USA) and signals were visualized and quantified using the Quantity One application (Bio-Rad, USA). The gray analysis was based on Image J software version 1.44p (NIH, USA).

### Semi-quantitative and quantitative RT–PCR

Total RNA was isolated using the RNA Extraction Kit (RP1202, BioTeke, Beijing, PRC) according to the manufacturer's instructions, and complementary DNA was made using First Strand complementary DNA Synthesis Kit (NP100042, OriGene) and HiScript II Q RT SuperMix for quantitative PCR(R222-01, Vazyme). Semi-quantitative RT–PCR was performed with Taq DNA Polymerase (Invitrogen). Quantitative real-time polymerase chain reaction (RT–PCR) was performed based on iCycle System (Bio-Rad) and The SensiMix SYBR Master Mix (QP100005, OriGene). PCR data were analyzed using Graph Pad Prism (Graph Pad Software, San Diego, California, USA). Information about the primers and probes used in quantitative PCR is in the Supplementary Table 7.

### Promoter-reporter assays

To verify that PHF8 is upregulated by HIFs, 293FT cells were co-transfected with plasmids containing either a control promoter or full PHF8 promoter (Supplementary Table 8) and plasmids expressing either HIF1α or HIF2α. Cells transfected with empty vectors served as negative controls. To verify that PHF8 transactivates the AR, HeLa cells were co-transfected with plasmids expressing Gal4-AR, Gal4 luciferase (4 × UAS-TK-Luc), renilla luciferase (transfection control) and plasmids expressing either wild-type PHF8 (PHF8wt) or mutant PHF8 (PHF8mu). Transfected cells were cultured with or without R1881 for 48 h before luciferase assays were conducted with the Dual-Luciferase Assay Kit (Promega, Madison, WI, USA).

### Co-Immunoprecipitation

Flag-tagged PHF8 and GFP-tagged AR were co-expressed in HEK293FT cells in the presence or absence of DHT. Antibodies against Flag or GFP were used for immunoprecipitation and western blot assays. Briefly, after co-transfection of plasmids with flag-tagged PHF8 and GFP-tagged AR for 48 h, HEK293FT cells were lysed in RIPA buffer with a protease inhibitor tablet. Lysates were rotated at 4 °C for 20 min and then homogenized by passage through a 21-gauge syringe needle five times, followed by another 20 min rotation at 4 °C. Insoluble material was cleared from the lysates by centrifugation at 12 000 *g* for 15 min. A sample of the lysate was set aside for analysis by western blotting to assess expression of transfected DNA proteins. The remainder of the cleared lysates was divided into two equal portions. Antibodies against either Flag (TA50011-100, OriGene) or GFP (66002-1-Ig, Proteintech) was incubated with cell lysate overnight at 4 °C and precipitated with protein A/G-Sepharose (sc-2002, Santa Cruz, Biotechnology) for 2 h at 4 °C. The precipitates were washed four times with phosphate-buffered saline at 4 °C. The proteins were eluted from the beads in 50 μl 2 × SDS sample buffer by heating for 5 min at 80 °C and analyzed by western blotting. To determine whether AR can interact with endogenous PHF8, lysate from LNCaP cells as well as 22RV1 cells were precipitated with antibodies against either AR (ab108341, Abcam) or PHF8 (NB100-93314, Novus Biologicals) followed by western blotting with antibodies against PHF8 and AR.

### Immunofluorescence and confocal microscopy

LNCaP or PC-3 cells transfected with plasmid expressing Flag-tagged PHF8 Wt were incubated for 40 h and further treated by 1% hypoxia for 12 h at 37 ºC, followed by fixation with 4% paraformaldehyde, cells were permeabilized with 0.5% Triton X-100 in phosphate-buffered saline at room temperature for 20 min, and blocked in 10% goat serum for 30 min at 37 ºC. Flag-tag was stained with mouse anti-Flag monoclonal antibody (1:1000; F1804, Sigma-Aldrich, St Louis, MO USA) at 4 °C and goat anti-mouse antibodies conjugated to fluorescein isothiocyanate (FITC; 1:200; SA00003-1, Proteintech) for 1 h at 37 ºC. To observe the methylation level, rabbit anti-H3K9me1, H3K9me2 and H4k20me1 primary antibodies (Abcam, 1:1000) and goat anti-rabbit secondary antibodies conjugated to cyanine 3 (Cy3; 1:400; CW0159, CWBiotech, Beijing, PRC) were used. The nuclei were stained for 15 min with 40, 60-diamidino-2-phenylindole (DAPI, KGA215, KeyGen BioTECH) at 37 ºC, and images were obtained with confocal microscopy (LSM700; Zeiss, Oberkochen, Germany).

### Chromatin immunoprecipitation (ChIP) assay

Cells were cross-linked with 1% formaldehyde (Sigma) and chromatin DNA was extracted using the chromatin extraction kit (ab117152, Abcam). The DNA was sheared to 200–1000 bp by sonicating 4x10s at 40% of maximum force. IgG or antibody against PHF8 or AR were added to the lysates and incubated for 2 h at room temperate on an orbital shaker (100 r.p.m.). After washing four times, the cross-linked protein–DNA complex was reversed by proteinase K at 65 °C for 15–20 min, followed by incubation at 95 °C for 5–10 min. Purified DNA was used for PCR with specific primer pairs targeting the enhancer regions of the PSA and NKX3.1 promoter. Information of the primers was shown in the Supplementary Table 9.

### PHF8 siRNA transfection

VCaP cells were transfected with chemical systhesis siRNA against human PHF8 (Invitrogen). RT–PCR were used to detect PHF8 knockdown in VCaP cells. Information of the primers was shown in the Supplementary Table 10.

### Statistical analysis

All data are expressed as means±standard error. Comparisons between two groups were conducted using unpaired Student's *t*-test. Experiments with more than two groups were compared by one-way ANOVA with Tukey's multiple comparison for equal variances or by Kruskal–Wallis test followed by Dunn's Multiple Comparison if equal variances were not assumed. Statistical analyses were performed using Graph Pad Prism version 5 (Graph Pad Software, San Diego, Calif., USA). A value of *P*<0.05 was considered to be statistically significant. Kaplan–Meier plots with log-rank statistical testing and Cox regression analysis was used in patient survival analysis. Tests were undertaken using SPSS, version 19.0, computer software (SPSS Inc., Chicago, IL, USA).
