# Supplementary material for: The HIF/PHF8/AR axis promotes prostate cancer progression
Source: Oncogenesis. 2016 Dec 19;5(12):e283–. doi: 10.1038/oncsis.2016.74 (PMC5177772; doi:10.1038/oncsis.2016.74)
Supplement: Supplementary Information [file oncsis201674x1.pdf]

Figure S1

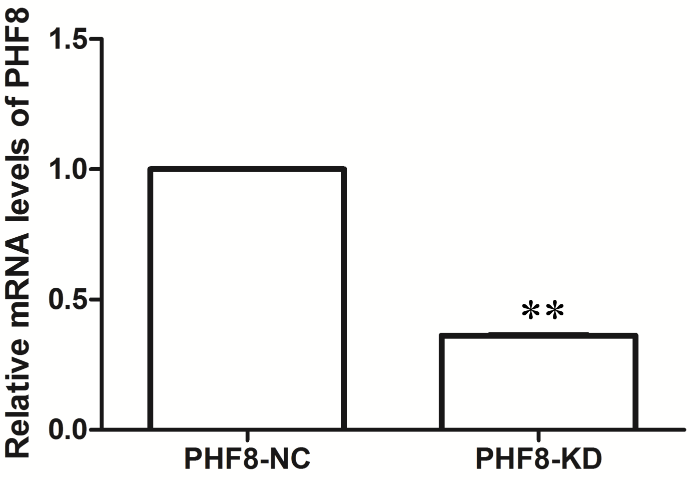

Figure S1. LNCaP cells were infected with a lentiviral shPHF8 and qPCR used to estimate the efficiency of PHF8 knockdown.

Figure S2

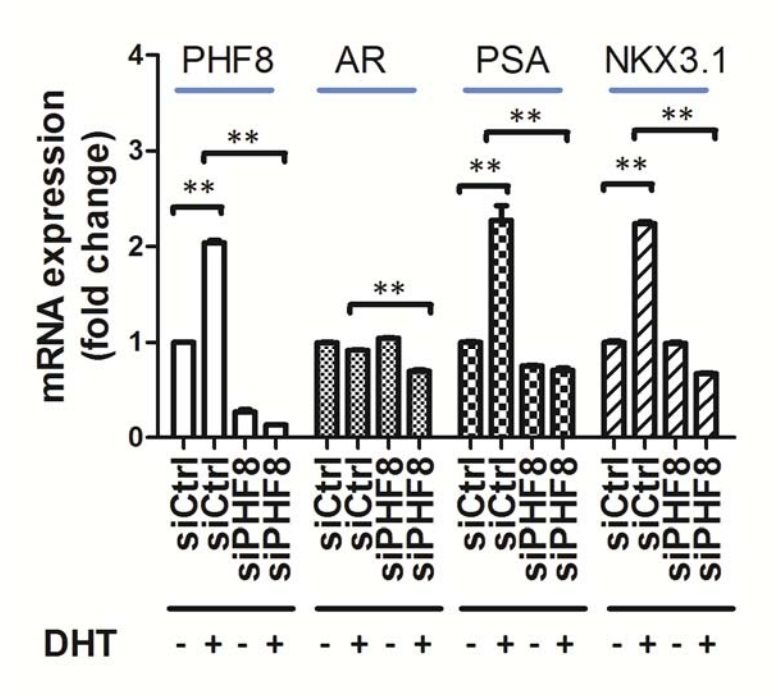

Figure S2. Knockdown PHF8 severely impaired DHT-induced transcriptional activation of PSA and NKX3.1. The control siRNA and siPHF8 transfected VCaP cells were treated with 10 nM DHT for 24 hours and total RNAs were prepared and subjected to quantitative RT-PCR analysis.

Figure S3

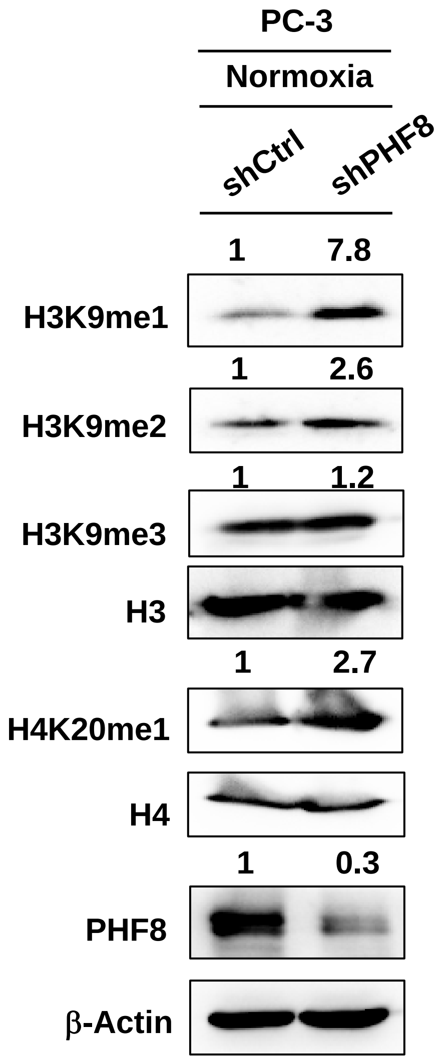

Figure S3. PC-3 cells were stably infected with control shRNA or shPHF8 and the histone methylation status under normoxic condition were analyzed by western blot.

Figure S4

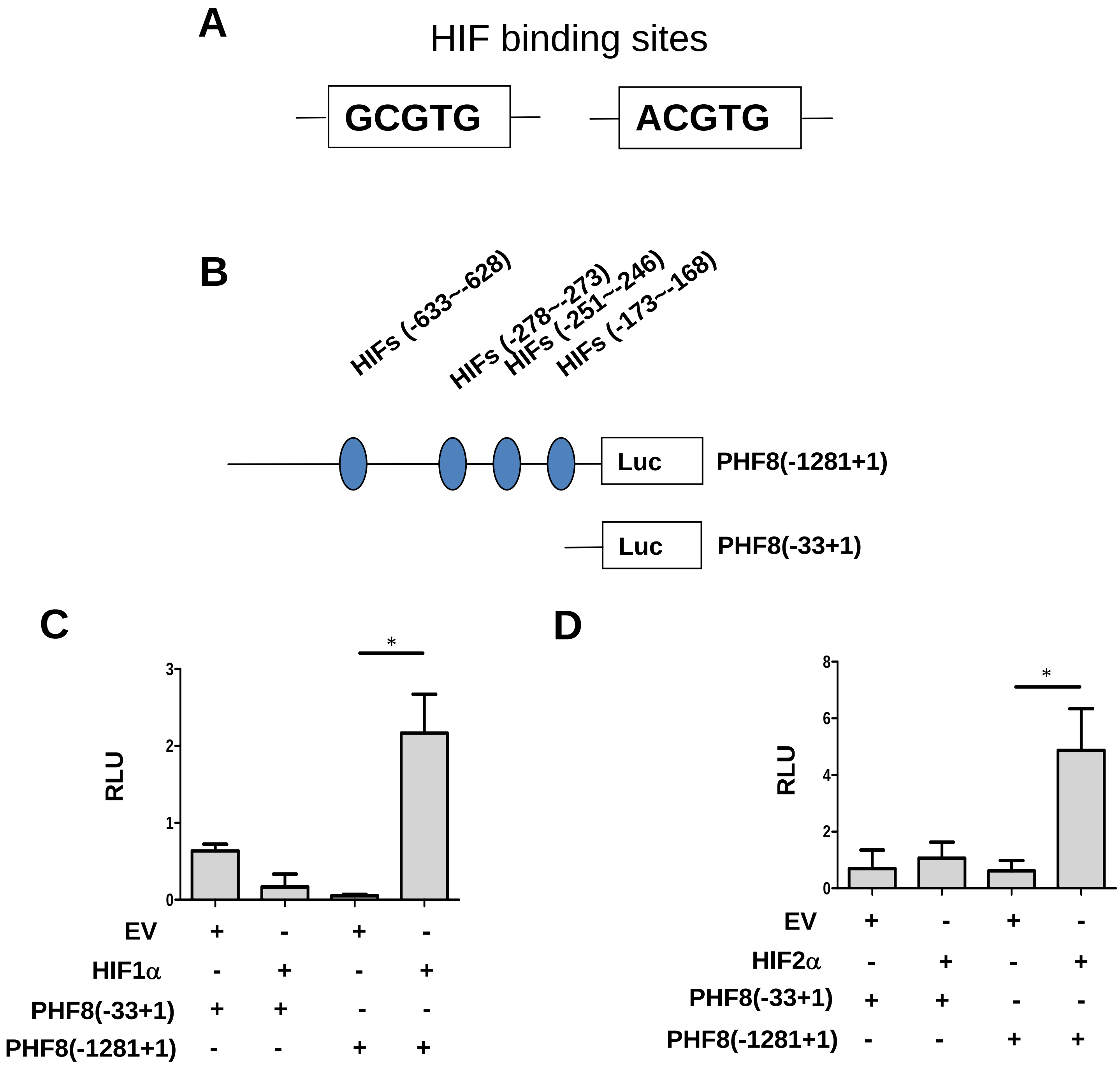

Figure S4. PHF8 is directly activated by HIF transcription factors. (A) HIF binding sites (B) Schematic structures of PHF8 full length promoter sequence with HIFs-binding sites were shown. (C) and (D) 293FT cells were transfected with plasmid encoding either control promoter or PHF8-full length promoter together with plasmid expressing either HIF1α (C) or HIF2α (D). Luciferase assays were performed to analyze transcriptional activation of PHF8 by HIFs.

Figure S5

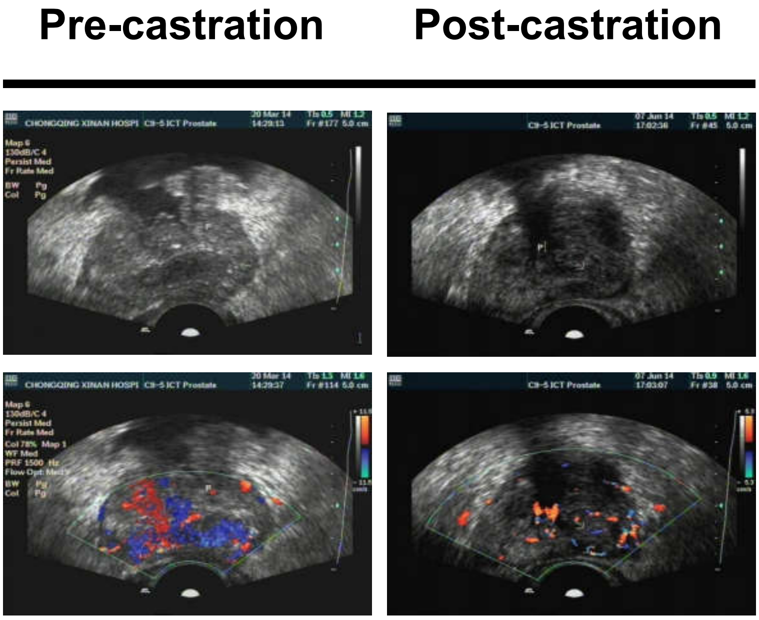

Figure S5. Representative images of color doppler imaging of transrectal ultrasound for prostate cancer tumor of the same patients pre-and post-castration treatment.

Figure S6

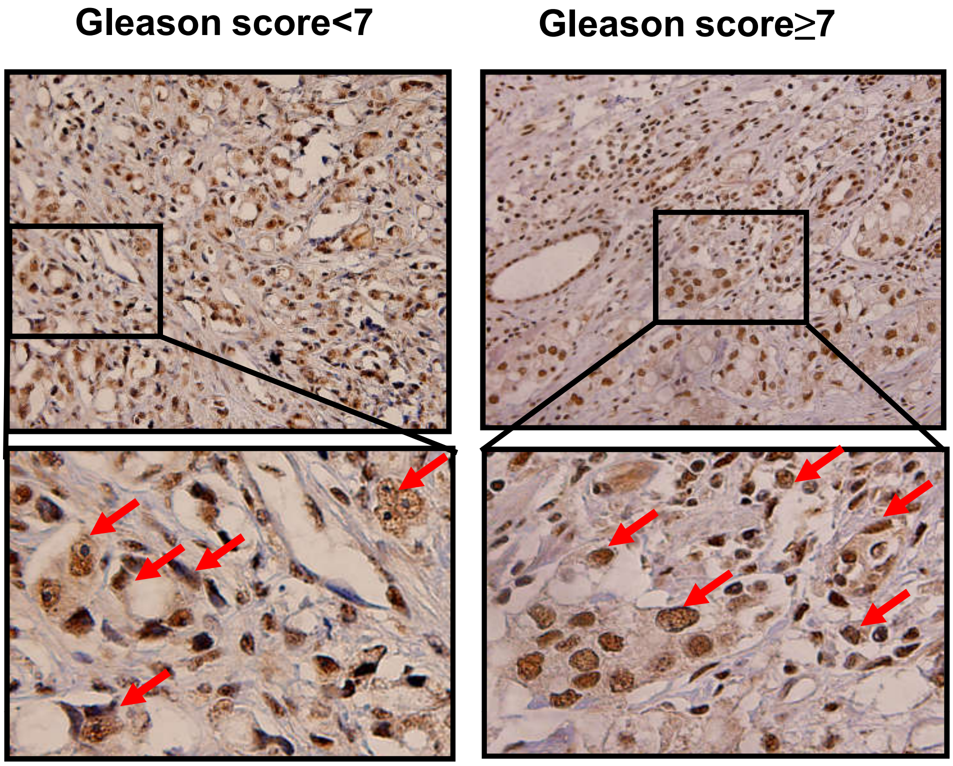

Figure S6. The representative results of immunohistochemistry analysis on PHF8 expression in clinical samples with Gleason<7 and Gleason≥7. Original magnification was ×400 (upper panel) and higher magnification images shown in lower panel. The clinical samples with Gleason<7 is predominant in cytoplasm staining location (lower left panel indicated by red arrows) and those with Gleason≥7 predominant in nuclear staining location (lower right panel indicated by red arrows).

Table S1. The patients' clinical characteristics and expression levels of HIF1α, HIF2α and PHF8 in the prostate cancer tissues before and after castration treatment

		Pre-castration					Post-castration			
PID	Age	Gleason	HIF1α	HIF2α	PHF8	ADT time(m)	Gleason	HIF1α	HIF2α	PHF8
1	59	3+4	+	++	+++	1	3+4	+++	+++	+++
2	70	3+4	+	++	++	3	3+3	+++	+++	+++
3	67	2+3	+	++	++	1	2+3	+++	+++	+++
4	53	3+3	+	++	+	2	5+3	++	++	+++
5	72	3+4	++	++	++	1	3+4	++	+++	+++
6	73	4+5	+	++	++	3	5+5	++	+++	+++
7	67	3+3	++	++	+++	3	4+3	++	+++	+++
8	74	3+4	+	+++	+++	1	3+4	+++	+++	+++
9	70	3+3	++	++	++	1	3+4	+++	+++	+++
10	71	5+3	++	++	+++	1	4+5	+++	+++	+++
11	69	4+3	+	+	+	1	5+4	++	+++	+++
12	65	3+3	++	+++	++	1	4+5	++	+++	+++
13	65	3+3	+	++	+++	1	3+4	++	+++	+++
14	65	5+3	+++	+++	+++	1	5+3	+++	+++	+++

The HIF1α, HIF2α and PHF8 levels were estimated by IHC staining and scored (Materials and Methods). The IHC score of 1~2 was labeled as "+", 3~4 as "++", and 6~9 as "+++". PID, Patient ID. ADT (m): Androgen deprivation therapy (month).

Table S2. The expression of HIF1α, HIF2α and PHF8 in patients pre- and post-castration treatment

	HIF1α Expression		*P*-Value	HIF2α Expression		*P*-Value	PHF8 Expression		*P*-Value
	Weak	Strong		Weak	Strong		Weak	Strong	
Pre-castration	13	1	0.033	11	3	<0.001	8	6	0.003
Post-castration	7	7	*	1	13	***	0	14	**

Pearson $\chi 2$ tests were used to assess statistical significance between groups. Significant differences in HIF1α, HIF2α and PHF8 expression between pre- and post-castration groups are denoted by ***， $P<0.001$, **, $P<0.01$, *, $P<0.05$.

Table S3. The patients' clinical characteristics and relationship between the levels of PHF8 expression and survival time were shown

PID	Age	Gleason	PHF8 IHC score	Survival status 0: Live 1:Dead 2:Censored	Survival months
1	60	5+4	3*2	1	32
2	59	4+3	2*2	0	94
3	73	3+5	3*2	1	4
4	58	4+5	3*2	1	60
5	65	4+3	3*1	0	64
6	57	4+5	3*2	1	54
7	59	5+4	3*3	2	66
8	62	4+4	3*3	2	104
9	70	5+3	3*3	0	93
10	72	4+5	3*3	1	54
11	56	3+3	2*2	2	96
12	69	4+3	2*2	0	56
13	73	3+5	2*3	2	76
14	70	2+3	2*1	0	75
15	58	4+3	3*3	1	34
16	73	4+5	3*3	1	27
17	74	2+4	3*2	2	6
18	63	3+3	2*2	2	60
19	72	5+4	3*3	2	61
20	74	5+3	3*3	1	65
21	65	4+3	3*1	1	13
22	61	3+4	3*2	2	26
23	67	5+3	3*2	1	84
24	70	3+4	1*2	1	61
25	71	5+4	3*3	1	54
26	73	3+4	2*3	1	65
27	73	3+5	2*2	2	69
28	70	3+3	3*3	0	59
29	73	4+5	3*2	2	50
30	66	5+3	1*2	0	93
31	56	5+4	2*2	1	41
32	70	4+3	2*3	0	69
33	69	4+5	3*3	1	70
34	63	3+4	3*2	1	38
35	68	3+3	3*2	0	65
36	69	2+3	1*2	2	104
37	63	4+3	3*1	1	42
38	73	5+3	3*3	1	58
39	71	3+3	2*2	0	70
40	61	3+3	3*1	1	96
41	59	3+2	2*3	1	51
42	73	4+3	2*2	0	116
43	74	4+3	1*1	1	44
44	55	5+4	2*2	2	66
45	73	3+3	2*2	1	76
46	66	2+4	3*1	2	36
47	65	3+4	2*2	2	73
48	68	3+4	1*2	0	62
49	69	4+3	2*2	0	86
50	70	3+5	1*2	1	76
51	71	2+1	2*2	1	63
52	65	3+3	2*2	2	32
53	44	5+3	3*3	1	22
54	73	5+5	3*3	1	32
55	71	5+3	3*2	0	74
56	57	3+4	1*3	0	86
57	63	3+3	2*2	1	73
58	73	4+3	3*3	0	61
59	73	4+3	2*2	1	65
60	67	4+3	3*3	0	65
61	68	3+3	1*2	0	118
62	69	3+2	1*2	2	18
63	56	2+3	2*1	0	111
64	67	4+3	2*3	0	64
65	59	5+4	3*3	1	53

PHF8 levels were estimated by IHC staining and scored (Materials and Methods).
PID: Patient Identification.

Table S4. The relationship between PHF8 expression and tumor-related variables were shown.

Variable	PHF8 Expression Weak	Strong	*P*-Value
Number	33	32	
Age,Y			0.710
⩽59	6	7	
60-75	27	25	
Gleason			<0.001
3-6	15	3	***
7	13	8	
8-10	5	21	

Pearson $\chi 2$ test was used to assess the statistical significance, and significant difference of PHF8 expression in different Gleason grading based on sample size calculation were shown as ***，P<0.001.

Table S5. PHF8 expression in CRPC vs castration-sensitive prostate cancer was shown.

Samples	PHF8 expression intensity		
	+++	+~++	-
Castration-sensitive	3	8	0
Castration-resistant	12	4	0
BPH	0	2	4

The numbers of patients with different levels of PHF8 in BPH, castration-sensitive prostate cancer and castration-resistant prostate cancer were shown. The difference of PHF8 expression intensity among the three groups based on sample size calculation was examined by Pearson $\chi 2$, $P<0.001$.

Table S6. The HIF1α, HIF2α (EPASα) and PHF8 shRNA sequences in Lentivirus

shRNA	Primer sequences				
HIF1-sh37-a	Ccgg	CCAGTTATGATTGTGAAGTTA	CTCGAG	TAACTTCACAATCATAACTGG	TTTTTg
HIF1- sh37-b	aattcaaaaa	CCAGTTATGATTGTGAAGTTA	CTCGAG	TAACTTCACAATCATAACTGG	
HIF1- sh39-a	Ccgg	GTGATGAAAGAATTACCGAAT	CTCGAG	ATTCGGTAATTCTTTCATCAC	TTTTTg
HIF1- sh39-b	aattcaaaaa	GTGATGAAAGAATTACCGAAT	CTCGAG	ATTCGGTAATTCTTTCATCAC	
HIF1- sh40-a	Ccgg	CGGCGAAGTAAAGAATCTGAA	CTCGAG	TTCAGATTCTTTACTTCGCCG	TTTTTg
HIF1- sh40-b	aattcaaaaa	CGGCGAAGTAAAGAATCTGAA	CTCGAG	TTCAGATTCTTTACTTCGCCG	
HIF1-sh41-a	Ccgg	TGCTCTTTGTGGTTGGATCTA	CTCGAG	TAGATCCAACCACAAAGAGCA	TTTTTg
HIF1-sh41-b	aattcaaaaa	TGCTCTTTGTGGTTGGATCTA	CTCGAG	TAGATCCAACCACAAAGAGCA	
EPAS1-sh60-a	Ccgg	gcGCAAATGTACCCAATGATA	CTCGAG	TATCATTGGGTACATTTGCGC	TTTTTg
EPAS1- sh60-b	aattcaaaaa	gcGCAAATGTACCCAATGATA	CTCGAG	TATCATTGGGTACATTTGCGC	
EPAS1- sh61-a	Ccgg	caGTACCCAGACGGATTTCAA	CTCGAG	TTGAAATCCGTCTGGGTACTG	TTTTTg
EPAS1- sh61-b	aattcaaaaa	caGTACCCAGACGGATTTCAA	CTCGAG	TTGAAATCCGTCTGGGTACTG	
EPAS1- sh62-a	Ccgg	cgACCTGAAGATTGAAGTGAT	CTCGAG	ATCACTTCAATCTTCAGGTCG	TTTTTg
EPAS1- sh62-b	aattcaaaaa	cgACCTGAAGATTGAAGTGAT	CTCGAG	ATCACTTCAATCTTCAGGTCG	
EPAS1-sh63-a	Ccgg	ccATGAGGAGATTCGTGAGAA	CTCGAG	TTCTCACGAATCTCCTCATGG	TTTTTg
EPAS1-sh63-b	aattcaaaaa	ccATGAGGAGATTCGTGAGAA	CTCGAG	TTCTCACGAATCTCCTCATGG	
PHF8-sh8-a	Ccgg	GGTGGAAGACATCTTCCAACA	CTCGAG	TGTTGGAAGATGTCTTCCACC	TTTTTg
PHF8- sh8-b	aattcaaaaa	GGTGGAAGACATCTTCCAACA	CTCGAG	TGTTGGAAGATGTCTTCCACC	
PHF8- sh9-a	Ccgg	GCTGGCCAGTTGAGCTATAAT	CTCGAG	ATTATAGCTCAACTGGCCAGC	TTTTTg
PHF8- sh9-b	aattcaaaaa	GCTGGCCAGTTGAGCTATAAT	CTCGAG	ATTATAGCTCAACTGGCCAGC	
PHF8- sh10-a	Ccgg	GCTCTTTCCAGAAAGCAAAGT	CTCGAG	ACTTTGCTTTCTGGAAAGAGC	TTTTTg
PHF8- sh10-b	aattcaaaaa	GCTCTTTCCAGAAAGCAAAGT	CTCGAG	ACTTTGCTTTCTGGAAAGAGC	
PHF8-sh11-a	Ccgg	GCAGAGTATATCTATCCTTCA	CTCGAG	TGAAGGATAGATATACTCTGC	TTTTTg
PHF8-sh11-b	aattcaaaaa	GCAGAGTATATCTATCCTTCA	CTCGAG	TGAAGGATAGATATACTCTGC	

Table S7. The primers used in quantitative PCR

Gene	qPCR-Primer sequences
PHF8	F: TTCATGGCAGTTGTGTTGGTG
	R: TGCAAGACTTCACAGTTGGGG
AR	F: GACGACCAGATGGCTGTCATT
	R: GGGCGAAGTAGAGCATCCT
PSA	F: GTGTGTGGACCTCCATGTTATT
	R: CCACTCACCTTTCCCCTCAAG
NKX3.1	F: CCCACACTCAGGTGATCGAG
	R: GAGCTGCTTTCGCTTAGTCTT
GAPDH	F: AAGGTGAAGGTCGGAGTCAAC
	R: GGGGTCATTGATGGCAACAATA

Table S8. The sequences of PHF8 full length promoter

AGATCTCCCAGCCTATCGAAGGTGCTCAATGAACAGGAGCCGCGGTGAGTACTCCGC
CTCTACCCCGGCTGAAGCCCGCCCCCGCCGCCACCTATTAATTTTGTAGTTTGGAGTC
ACCGAGTTCACCTCCTCTCATAGGAGGAGAAGGCTGGGGAGGAGGAGTGTGCGAGT
CGGGGTAGCTGAACTCCCTGTTTGCCCGGAGGAGGGGGAAGGCTAGGAAGTTTCGG
GCCCCACTCAAAATGGAGCCAAACGTCTGCCTCGCCTAAGATGGCGGCTTCTCCGCC
TCTGGGCTCAAGGTCTTCAGCGGCGATTGGGCACCTTAAAGAGACGAGCGGGTCCA
AAGGTGGCTGAGAACCGCCCTCCTTTCTCAAGGTGGCGCCCCGCCCCCCCGATGAC
GTCACCCGGGAGCCGGCGGCTCGCGGTTCCCCGCCTCCGCCGCGCTCACCTCGCGA
GTCCAGCCCTCATAAGATGGCGGTCGCCTTGGTTGTCTGGGTAGCGCCGCCACTCTC
AACATGGTAGCTGTTTATCCCGTATTTCTCCTCCCCTCTCCGCCTCAACTTCCTCATTGT
TTTGAATAAACTTTATTGACTACTCCGAATTGCCTACCACCGCCACTGGGCTTCTCCCA
GCGACTTTTCGGGCCGTGTGCCTCGGACGTGGCGGCCGGTTCATCTCTTTCCCCGTT
GGTTTGCTCGCGAGTATCTCCTGCCGGCCCAGCGACGACGCCACTTGCCCGTTCGCC
GGGCGGCCGCCAGACTAGGCCCAAGCCGCGGTCTCCAGTAGGCCCGAACGGCCGG
GCCGAGGGGAATGTTGTGGAGGAGGCTGCTCTGAAGCACCGTTGAGCGGCTGGCGC
CGCGCGACCCAGCGGGGGGCTCGAGGGGAAGGCGAGCGAGGTTCCCGGCGGTACG
GGGACTATCCCAGAATTCGACGCGCGTCGCCGTAGGGGCCGGAACTACCGGACGAG
CCTCCGCTGAGGCGCTTCGCAGTCCCGGAGCTAGCCCGGCTGCCGGCGTGTCGCTG
GGGCTGAGCTCCGCGGGCGTGGAGTCCTTGCAGCCCAAAGCATGAGGAGGTCCCTG
TAGGATTCTGGACTGAAGACGTTCTTGTCAGGTTTGGGGCGTGAGGAGGTTCCTGTC
AGTTGGGGAAGCGTTAAGATTCCTGTAAGTTTTGGGGGCTGCGAGGCTATTACTGTCA
GTTTTAGGATATACTGTGAGGTACCTGAACATTTGGGGAGACTAGGGAGCTCTCATCA
GTTTGAGGGGGGGCTGCAAGGAAACGCTGCCAGTTTCCAAGCTT

Table S9. The primers used in quantitative ChIP-PCR

Gene	ChIP-qPCR Primer sequences
PSA	F: CCTAGATGAAGTCTCCATGAGCACA
	R: GGGAGGGAGAGCTAGCACTTG
NKX3.1	F: GTAGTCTGTCTGGGAAAG
	R: TACTGCTCAAGAAAAAGGGC

Table S10. The PHF8 siRNA sequence

Gene	Primer sequences
PHF8	GCCUGCUGGCCAGUUGAGCUAUAAU
